# Meta-representations as representations of processes

**DOI:** 10.1093/nc/niaf038

**Published:** 2025-10-30

**Authors:** Ryota Kanai, Ryota Takatsuki, Ippei Fujisawa

**Affiliations:** Management, Araya, Inc., Sanpo Sakuma Building, 1-11 Kanda Sakuma-cho, Chiyoda-ku, Tokyo 101-0025, Japan; Research & Development Department, Araya, Inc., Sanpo Sakuma Building, 1-11 Kanda Sakuma-cho, Chiyoda-ku, Tokyo 101-0025, Japan; Graduate School of Interdisciplinary Information Studies, The University of Tokyo, 7 Chome-3-1 Hongo, Bunkyo-ku, Tokyo 113-8654, Japan; Sussex Centre for Consciousness Science, Department of Engineering and Informatics, University of Sussex, Falmer, Brighton, BN1 9QT, England; President’s Office, Araya, Inc., Sanpo Sakuma Building, 1-11 Kanda Sakuma-cho, Chiyoda-ku, Tokyo 101-0025, Japan

## Abstract

In this study, we explore how the notion of meta-representations in higher-order theories (HOT) of consciousness can be implemented in computational models. HOT suggests that consciousness emerges from meta-representations, which are representations of first-order sensory representations. However, translating this abstract concept into a concrete computational model, such as those used in artificial intelligence, presents a theoretical challenge. For example, a simplistic interpretation of meta-representation as a representation of representation makes the notion rather trivial and ubiquitous. Here, as a foundational step toward understanding meta-representations, we propose a refined computational interpretation that focuses specifically on process-level representations. Contrary to the simplistic view of meta-representations as mere transformations of the first-order representational states or confidence estimates, we argue that meta-representations represent the computational processes that generate first-order representations, building on the Radical Plasticity Thesis by Cleeremans (2011). https://www.frontiersin.org/journals/psychology/articles/10.3389/fpsyg.2011.00086.) This presents a process-oriented view whereby meta-representations capture the qualitative aspect of how sensory information is transformed into first-order representations. As a proof-of-concept of this formulated notion of meta-representation, we constructed "meta-networks" designed to explicitly model meta-representations within deep learning architectures while methodologically isolating process representations from specific sensory activations to avoid confounding effects. Specifically, we constructed meta-networks by implementing autoencoders of first-order neural networks. In this architecture, the latent spaces embedding those first-order networks correspond to the meta-representations of first-order networks. By applying meta-networks to embed neural networks trained to encode visual and auditory datasets, we show that the meta-representations of first-order networks successfully capture the qualitative aspects of those networks by separating the visual and auditory networks in the meta-representation space. We argue that such meta-representations would be useful for quantitatively comparing and contrasting the qualitative differences of computational processes. While whether such meta-representational systems exist in the human brain remains an open question, this formulation of meta-representation offers a new empirically testable hypothesis that there are brain regions that represent the processes of transforming a representation in one brain region to a representation in another brain region. Furthermore, this form of meta-representations might underlie our ability to describe the qualitative aspect of sensory experience or qualia.

## Introduction

Theories of consciousness offer insights into the functional basis of consciousness (Seth and Bayne 2022). However, while they make intuitive appeal, the theoretical concepts introduced by such theories still leave some ambiguity when we attempt to translate the theoretical frameworks into computational models. For example, consider the components of the global workspace theory (GWT), such as broadcasting of information across a network of specialized modules (Baars 1993, Dehaene et al. 1998, Baars et al. 2021). At a conceptual level, the GWT elegantly captures how disparate pieces of information become part of a cohesive conscious experience, and offers possible interpretations of neural activations associated with conscious processing (Mashour et al. 2020). However, the actual implementation of such a global workspace within a computational model leaves ambiguities, such as what constitutes the global workspace and what it means to broadcast information. Another example is the higher-order theories (HOT) of consciousness (Rosenthal 2005, Lau and Rosenthal 2011). These theories propose that a mental state becomes conscious by virtue of meta-representations, which are representations of representations. This meta-representation makes intuitive sense as it captures the general idea about awareness. However, operationalizing this concept in computational models such as deep neural networks raises questions as to what exactly a meta-representation is.

Deep learning offers a toolkit with potential applications in modeling neuronal processes underlying consciousness (Juliani et al. 2022a, 2022b, Butlin et al. 2023, Zador et al. 2023). Neural network architectures with their multi-layered processing provide an analogy to the brain’s hierarchical information processing (Kriegeskorte 2015, Lindsay 2021). While there are admittedly fundamental differences, representation learning in neural networks makes a benchmark model of the representations in the brain (Nonaka et al. 2021). Attention mechanisms in the transformer architecture (Vaswani et al. 2017) also provides a concrete method to implement both bottom-up and top-down attention mechanisms, which are important ingredients of models of consciousness. The notion of latent space is particularly interesting as it provides concrete terms to talk about the rich compressed representations that constitute the conscious contents in the brain. Autoencoders and their variants (Hinton and Salakhutdinov 2006, Kingma and Welling 2013, Higgins et al. 2016), which consist of an encoder and a decoder, learn to compress high-dimensional sensory inputs into compact embedding vectors in an unsupervised manner. The resulting latent space has the property that Euclidean (or other) distances between points reflect similarity between the original stimuli. These deep learning tools collectively enable the re-interpretations of the functional mechanisms associated with theories of consciousness and provide tangible means to refine theoretical concepts in consciousness studies.

As an example of this approach, we have re-interpret global workspace as a shared latent space across specialized modules (Van Rullen and Kanai 2021). Such a constructivist approach facilitated understanding of functional advantages of possessing global workspace, and enabled testing of functional benefits in various forms of artificial neural networks (Goyal et al. 2021, Goyal and Bengio 2022, Juliani et al. 2022b, Sun et al. 2023).

In this paper, we discuss a possible re-interpretation of HOT from a deep learning perspective. In our re-interpretation, we argue that meta-representations represent first-order processes rather than simple transformations of first-order representations. As a proof-of-concept of this idea, we developed neural networks that take first-order neural networks as the inputs and embed them in the latent space. By applying the meta-networks to diverse first-order neural networks trained to encode visual and auditory datasets, we show that the meta-representations successfully capture the qualitative aspects of those networks by separating the visual and auditory networks in the meta-representation space. After showing the computational experimental results, we discuss possible implications of our hypothesis for biological brains. Finally, we will present our speculation about the possible link between meta-representations and qualia.

### Higher-order theories of consciousness

In consciousness research, there are two contrasting hypotheses regarding the conditions under which consciousness occurs. Here, we will call the two positions “first-order representation theory” and "higher-order representation theory". The first-order representation theory posits that the state of a system representing sensory information is sufficient for generating conscious experience of the sensory information. The idea is that only the state of representing sensory information is sufficient. That is, the subjective experience of seeing can be obtained simply by processing visual information. However, the first-order representation theory needs to answer the question of why some sensory processing does not give rise to conscious experiences. A possible answer to this question from the higher order representation theory is that first-order representation of sensory information is not enough for conscious experiences to occur, but a meta-representation of the first-order representation is required. While detailed discussion of various flavors of HOT is beyond the scope of the current paper (Rosenthal 2005, Lau and Rosenthal 2011), the common idea is that consciousness is associated with meta-representations that represent first-order representations. However, what exactly is meant by that meta-representation becomes a challenge when one tries to implement meta-representation in AI systems.

### Representation of representation

A simple interpretation of a meta-representation is to regard meta-representation as a representation of another representation, which can be obtained by some transformation. However, in the context of neural networks, if every layer in a neural network acts as a meta-representation of the previous one, then every transformation within the network could be considered a meta-representation. This widespread attribution dilutes the concept's explanatory power in relation to consciousness. Meta-representation, as postulated by HOT, should have a distinct quality that is not ubiquitously applicable to every stage of information processing within neural networks. The interpretation that any transformation can be considered a meta-representation raises concerns, potentially attributing a form of consciousness to almost every transformation within a neural network. However, most consciousness researchers would be reluctant to attribute consciousness to any hierarchical computation, as it would undermine the distinctive nature of conscious experience and its emergence from specific types of information processing.

**Figure 1:**
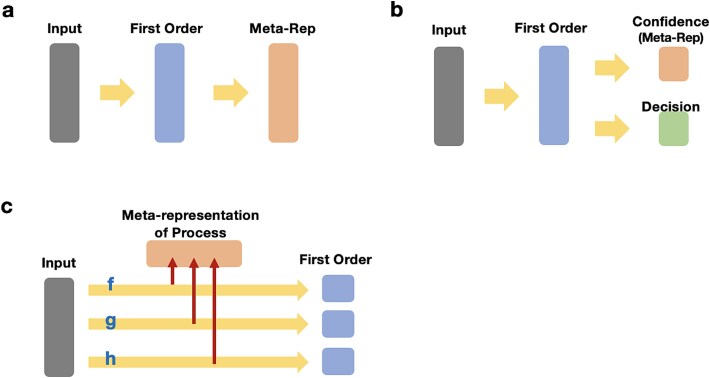
Three possible interpretations of meta-representation. a. A simplistic view of meta-representation where meta-representation is a representation of a first-order representation. b. Meta-representation as uncertainty/confidence estimation, where the first-order representation is used to compute the uncertainty of a decision. c. Meta-representation as a representation of a process transforming the input into the first-order representation.

This issue is illustrated in Fig. 1a. Let us consider a mapping *f*: *x* → *y* that transforms the input *x* to the output *y* by a neural network. Here, we simply define *y* as a representation of *x* for the sake of illustration. Next, we consider a map *g*: *y* → *z* that transforms *y* to *z* by another neural network. Then, *z* is a representation of the representation *y*. The question is whether we should consider *z* a meta-representation of *x*? However, in this simplistic case, *z* is just a first-order representation that is obtained by transforming *x* with the map $g\circ f$. This does not yield a qualitative difference to the representation of a representation, because it is just another representation. The issue of formulating meta-representations this way has previously been brought up by Cleeremans, who suggested that higher-order representations should be separated from the causal chain of the first-order networks (Cleeremans 2011).

### Uncertainty estimation as meta-cognition

In cognitive neuroscience of consciousness, experiments on metacognition are widely conducted by asking participants to report their confidence about whether their responses in a task are correct (Fleming et al. 2010, Kanai et al. 2010, Sandberg et al. 2010, Maniscalco and Lau 2012, Fleming and Lau 2014, Sherman et al. 2015, Fleming et al. 2023). Confidence rating is important as a signature of conscious perception, since many tasks (e.g. visual discrimination tasks) are known to be performed even without awareness (Cowey and Stoerig 1991, Kolb and Braun 1995, Lau and Passingham 2006). As such, correspondences between first-order objective performance (i.e. whether the judgement about the stimulus is correct or not) and confidence rating (i.e. confident *versus* not confident) are taken as a proxy measure for conscious perception. Thus, confidence levels associated with performance in tasks are often regarded as indicators of awareness.

The ability to estimate uncertainty could, therefore, be seen as a form of meta-cognition of one's own cognitive processes and has often been discussed in the context of HOT. Computationally speaking, confidence rating is an uncertainty estimation about the signals needed for the first-order decision. Thus, while confidence rating has been used in psychological experiments, it can be relatively simply implemented in AI systems (Fig. 1b). In a neural network, confidence is simply the estimation of uncertainty about its predictions. The softmax function is commonly used in deep learning to estimate certainty or probability distribution over a set of outcomes (Guo et al. 2017). This can be seen as a mechanism to compute confidence in sensory decision-making. However, this statistical confidence does not necessarily equate to the richer concept of meta-representation described by HOT of consciousness. The softmax function lacks the “aboutness” characteristic of conscious states. Given the simplicity of computing confidence in AI systems, it is unclear whether confidence counts as a meta-representation.

These considerations illustrate that formulating meta-representation in terms of neural networks is not straightforward and requires a refinement of the concept for implementation. In what follows, we propose a different approach by considering a possible AI implementation of meta-representations.

### Meta-representation as representation of process

We propose reframing the concept of meta-representation as a representation of the computational process itself. This process-oriented view is not the only alternative to simplistic interpretations, but it offers a theoretically grounded approach, building on established foundations in the literature, as we discuss later, that can be computationally implemented and empirically tested. This shift of the focus from representational states to processes makes the notion of meta-representation non-trivial and implies possible functional benefits of having meta-representations in conscious systems.

In this light, neural networks are considered as functions f(x), g(x), and h(x) mapping sensory data to internal, first-order representations (Fig. 1c). By regarding those processes as data (e.g. the joint distributions of the input and the output or the weight structure of the networks), functions can be embedded into meta-representations M(f), M(g), and M(h) of these mapping processes. With this formulation of meta-representations, we obtain an embedding space of neural networks where comparisons of different networks are possible. This is analogous to semantic embeddings of words as in Word2Vec (Mikolov et al. 2013), where the high-dimensional space captures semantic relationships among different words in natural language. Thus, the meta-representations allow for qualitative comparisons of specialized neural networks in terms of their functional similarities.

Note that formulating meta-representation as a representation of input and output relationship is not entirely new. Cleeremans *et al.* have extensively explored similar conceptualizations of meta-representations in the context of modeling metacognition and consciousness (Cleeremans et al. 2007, Pasquali et al. 2010, Cleeremans 2011, 2019, Timmermans et al. 2012). Notably, Cleeremans' approach offers distinctive cases where second-order networks take both the input and the output of the first-order network to represent the process itself, rather than merely focusing on activation patterns or confidence estimates. As such, our core proposal of taking representation of processes as meta-representations has been discussed in a series of works by Cleeremans *et al.*

Of particular note is the Radical Plasticity Thesis by Cleeremans (2011), which posits that consciousness arises from the brain's capacity to learn about its own representations through continuous redescription—a process termed representational redescription (Clark and Karmiloff-Smith 1993).

While the previous computational implementations focused on monitoring the performance of the first-order networks, i.e. the confidence, Cleeremans had already proposed that the second-order networks can capture the qualitative aspects of the first-order processes, going beyond simple monitoring of confidence levels (Pasquali et al. 2010, Timmermans et al. 2012).

In their computational models, Cleeremans *et al.* implemented second-order networks that access both the inputs and outputs of first-order networks to model the transformation processes. By taking into account the input–output relationships, these second-order networks can form representations of the first-order processes in addition to those of the first-order representations. This decomposition allows for separate analysis of process-level and representation-level contributions to meta-representations.

Building upon this foundational work, we aim to clarify the concept of meta-representation by focusing on representing the qualitative aspects of the first-order processes themselves. While previous models have primarily addressed the monitoring and evaluation of first-order representations—such as estimating confidence or uncertainty (Pasquali et al. 2010; Timmermans et al. 2012)—our approach seeks to capture the inherent qualities of different sensory modalities and tasks by embedding first-order networks into a latent space using their connectivity patterns (i.e. weight matrices).

This perspective allows us to compare the qualitative differences between networks trained on different modalities, potentially providing a computational framework for understanding qualia—the subjective qualities of conscious experience. By representing the processes that generate first-order representations, rather than just the representations themselves or their confidence levels, we aim to operationalize the concept of meta-representation in a way that captures the “aboutness” characteristic of conscious states (Cleeremans 2011).

Our work is motivated by the idea that consciousness involves not only being aware of one's own cognitive states but also understanding the nature of the processes that give rise to these states (Cleeremans 2019). By embedding first-order networks in a meta-representation space, we can explore how different sensory experiences relate to one another and how the brain might represent these relationships. This approach extends the concept of representational redescription and offers new insights into how meta-representations could be implemented in artificial neural networks, potentially shedding light on the neural mechanisms underlying consciousness.

In what follows, we show how this form of meta-representation can be implemented in artificial neural networks, and show that thus obtained meta-representations capture the features of first-order neural networks trained on various types of visual and auditory stimuli.

## Methods

### Meta-representation and meta-network

Following the reasoning we discussed so far, we consider representations of processes as meta-representations. To obtain concrete examples of meta-representations, we use what we call “meta-networks”, which take neural networks as the input or the output. That is to say, meta-networks are neural networks that take the weights of neural networks as input and embed those artificial neural networks into a latent space. By doing so, meta-networks learn low-dimensional representations of the diverse neural networks and provide insights into their abilities by allowing for the comparison and interpretation of networks trained on different datasets. A neural network that takes another neural network as input and classifies it into some categories (e.g. vision model or audio model) can be called a meta-classifier. In what follows, we provide a concrete example of meta-autoencoder and low-dimensional representations of first-order neural networks trained on various visual or auditory tasks.

Before detailing our specific hypothesis, it is crucial to clarify the rationale for this design. Our central goal is to test whether the computational process itself, or a network’s weights, acquires an intrinsic qualitative character. A meta-representation that has access to both the process (i.e. the first-order network's weights) and its input-dependent state (i.e. the first-order network's activations) would be the most expressive, capable of capturing fine-grained phenomenal details. However, such a design would obscure the contribution of the process with the influence of the input statistics. To methodologically isolate the process, our meta-networks were therefore designed to take only the first-order network’s weights as input. It is important to note, however, that this model is not entirely input-independent. We assume that the input to the meta-network is a first-order network that becomes highly activated by a sensory event. Thus, while the meta-representation is independent of the specific content of the input-state, it is contextually selected by the input.

### Hypothesis

Our hypothesis is that meta-networks can be trained to learn representations of first-order neural networks that capture the qualitative nature of the first-order neural networks. More specifically, we predict that the embedding of neural networks will exhibit that networks trained on similar tasks are clustered together, while those trained on different tasks are separated. Furthermore, this meta-representation should allow for the prediction of novel neural networks as to what kind of tasks they were trained for just by inspecting the weight patterns of the trained networks.

To test this hypothesis, we adopted multiple datasets for two modalities, vision and audio, and trained many first-order neural networks for each dataset. Using this model dataset, we trained a meta-autoencoder. Then, we fit a k-Nearest Neighbor (KNN) classifier to predict on which dataset a first-order neural network was trained based on their meta-representations embedded by the encoder part of the meta-autoencoder. Using this meta-classifier, we evaluated the prediction accuracy of the test datasets, which were not included in the training.

### Model architecture

**Figure 2:**
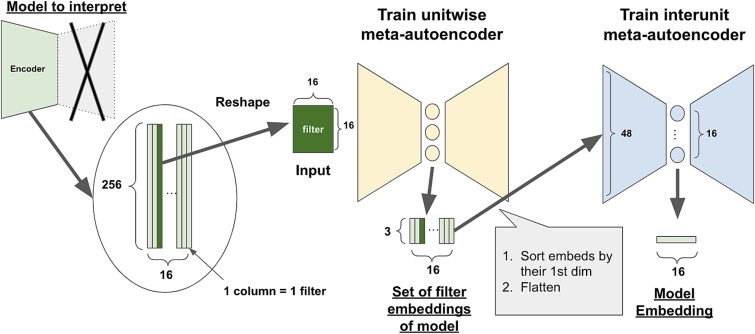
Overall structure of the construction of meta-autoencoders. Individual first-order networks are autoencoders trained on a single class from one of the datasets. The encoder part consisted of two fully connected layers, namely, the first layer consisting 256 units and the intermediate layer consisting of 16 units. The weight matrix from the first layer to one of the units in the intermediate layer comprised a filter represented by a 256-dimension vector. The filters were reshaped to a 16 × 16 matrix, which was used as the input to the unitwise meta-autoencoder, which further compresses the filters to a 3-dimensional vector in the latent space. Then, we repeated this for all 16 filters to obtain a set of their latent representations. The set of vectors was flattened to make a 48-dimension vector to be used as the input to the interunit meta-autoencoder. The 16-dimensional representations in the latent space of the interunit meta-autoencoder were taken as meta-representations of the first-order networks.

The architecture of the first-order networks was the encoder part of an autoencoder, consisting of two fully connected layers (see the left part of Fig. 2). This autoencoder has 256 dimensions for the input layer and the output layer, and the intermediate layer receiving inputs from the 256 units has 16 dimensions. Thus, the first-order model consisted of a 256 × 16 weight matrix (the encoder part has no bias). For simplicity, we excluded the batch normalization and ReLU layers from the encoder part. We trained a total of 600 networks per dataset, 500 of which were used to train the meta-autoencoder and the meta-classifier, and 100 to validate the predictions of the meta-classifier. Weights were initialized with a Glorot uniform initializer (Glorot and Bengio 2010), the loss function was mean squared error, the number of epochs was set to 1, and Adam (Kingma and Ba 2014) was used as the optimizer.

### Datasets

Multiple datasets are used for the two modalities, vision and audio. In order to train models of the same architecture on datasets for both modalities, we formatted the data for each modality as described below.

For vision datasets, all 20 classes from Pascal VOC dataset (Everingham et al. 2010) were treated as separate vision datasets. 10 of those datasets were used to prepare the model dataset for the meta-autoencoder. For each dataset, 01-normalization and grayscaling are performed, and 1 000 000 patches of size 16 × 16 are randomly sampled, each then flattened to 256 dimensions. We refer to each dataset as “v:” followed by the first three letters of the dataset name (e.g. “v:aer” for “aeroplane” dataset in Pascal VOC dataset) in this paper.

For audio datasets, all 10 classes from Urbansound8k dataset (Salamon et al. 2014), and all of the 11 classes from IRMAS dataset (Bosch et al. 2012) were treated as separate audio datasets. Five datasets from both Urbansound8k and IRMAS dataset were used to prepare the model dataset for the meta-autoencoder. For each dataset, wave data is converted to mel-spectrogram with the number of frequency bins being 16, and after 01-normalization, 1 000 000 patches of size 16 × 16 are randomly sampled by moving a window of width 16 on the time axis, each then flattened to 256 dimensions. We refer to each dataset as “a:” followed by the first three letters of the dataset name (e.g. “a:air” for “air-conditioner” dataset from IRMAS dataset) in this paper.

### Two-stage architecture of meta-autoencoder: unitwise and interunit

To construct meta-representations of the first-order encoders, we took a two-stage approach. The first stage consisted of a “unit-wise” meta-autoencoder that autoencodes each column of weights (considered as a filter). The second stage consisted of an “inter-unit” meta-autoencoder that autoencodes the resulting set of embedded filter representations.

The unitwise meta-autoencoder reshapes a 256-dimensional filter to 16 × 16 first, compresses it to 3 dimensions through three convolutional layers and two full-connected layers, and then reconstructs it back to 256 dimensions. The interunit autoencoder first flattens 3 × 16 to 48 dimensions, compresses it to 16 dimensions, and reconstructs it to 48 dimensions. These two were trained in sequence due to the need for sorting the order filters, as we elaborate below. This two-stage approach to training meta-autoencoders is illustrated in Fig. 2.

The unitwise meta-autoencoder first reshapes a 256-dimensional filter to a 16 × 16 matrix. This matrix then undergoes a compression process through a series of three convolutional layers, each followed by batch normalization and max pooling. The data is then passed through two fully connected layers and compressed to a 3D embedding space. The decoder part mirrors the encoder part. ReLU is used as the activation function throughout the network, except for the output layer, where the linear activation function is used.

The interunit meta-autoencoder consists of two fully connected layers with a latent space of 16 dimensions. It first flattens 3 × 16 to 48 dimensions, compresses it to 16 dimensions, and reconstructs it to 48 dimensions. The linear activation function is used in both layers, along with the use of bias terms.

For both meta-autoencoders, the weights are initialized with a Glorot uniform initializer (Glorot and Bengio 2010), the loss function is mean squared error, the number of epochs is 50, and Adam (Kingma and Ba 2014) is used as the optimization function.

### Filter sorting

Since the order of the filters in a fully-connected layer of the first-order network is not essential for the model function, we should regard the 16 filters as a set. In this study, we simply sorted them in the first dimension. This filter sorting is conducted after the unitwise meta-autoencoder training as a preprocessing step for training the interunit meta-autoencoder. Our two-stage construction of meta-autoencoders was expected to absorb part of the order issues by embedding the filters in the first stage.

### Predicting the nature of the dataset from the embedding of first-order networks

A k-NN classifier (k = 10) was connected to the output layer of the meta-encoder with its parameters frozen. This compound model comprised a meta-classifier, which was trained to classify the dataset used for training the first-order neural network. During the training of the meta-classifier, the dataset IDs were used as the labels.

The meta-classifier was evaluated on two levels. The first evaluation was for predicting the modality of the model’s training dataset. This evaluation was conducted using 100 models for each of all 41 datasets, including those not involved in training the meta-autoencoder. The second evaluation was for predicting the training dataset ID of the first-order models. This only used the 20 datasets used for training the meta-autoencoder. To test whether obtaining a low-dimensional representation of the model actually facilitated the prediction, a k-NN classifier that took a first-order network’s flattened weight matrix (4096 dimensions) directly as input was also evaluated for comparison.

## Results

### Prediction of sensory modality

First, we tested whether the modality of the first-order networks could be predicted by the meta-classifier. The accuracy of modality prediction was 100% (4100/4100). This result indicates that even the modality of the task not included in the training set can be predicted with extremely high accuracy. Thus, the meta-representations we constructed capture the common characteristic specific to visual or auditory modalities.

**Figure 3:**
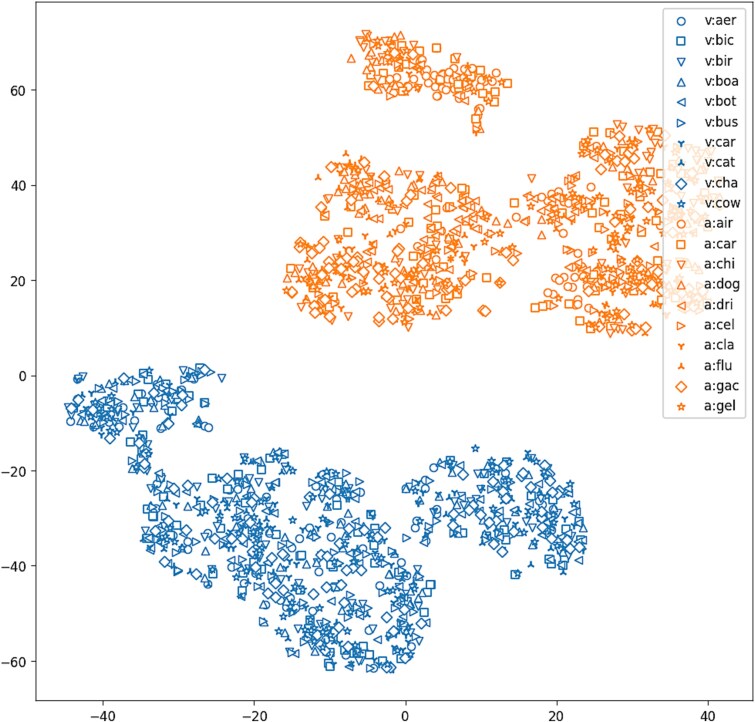
T-SNE visualization of first-order networks embedded by meta-autoencoder. Blue symbols (upper right) correspond to the networks trained on visual datasets, and orange symbols (lower left) to the networks trained on audio datasets.

To visualize the meta-representations of first-order networks, we applied t-SNE (Van der Maaten and Hinton 2008) (perplexity = 30) to the model vector representations of the validation datasets (Fig. 3). It can be seen that the two modalities are already clearly separated in this space, making it easy for the k-NN classifier to predict the modality. On the other hand, the prediction accuracy without using the meta-encoder was 50% (2050/4100). The breakdown was that the modality was predicted to be visual for all models. This implies that the weight space of the first-order models did not have a simple structure in terms of modality.

### Prediction of specific stimulus category

The accuracy of predicting specific dataset associated with a specific category of visual or auditory stimuli was 25.4% when meta-representations were used as the input. This is significantly higher than the chance level, which is 10%. This result should be interpreted in light of our methodology, which was designed to measure the expressive power of the process component in isolation. The fact that the accuracy is well above chance demonstrates that these process-level meta-representations contain meaningful and specific categorical information. On the other hand, the accuracy of the prediction without the help of the meta-encoder dropped to 7.85% indicating the importance of the meta-representations in capturing the characteristic features of first-order networks. Given that the task of each model is to encode a small region of the original dataset, the characteristic features within the same modality are likely to be similar to each other. Nevertheless, our current results suggest that our meta-autoencoder still captures subtle differences among the specific categories of the datasets within the same modality.

**Figure 4:**
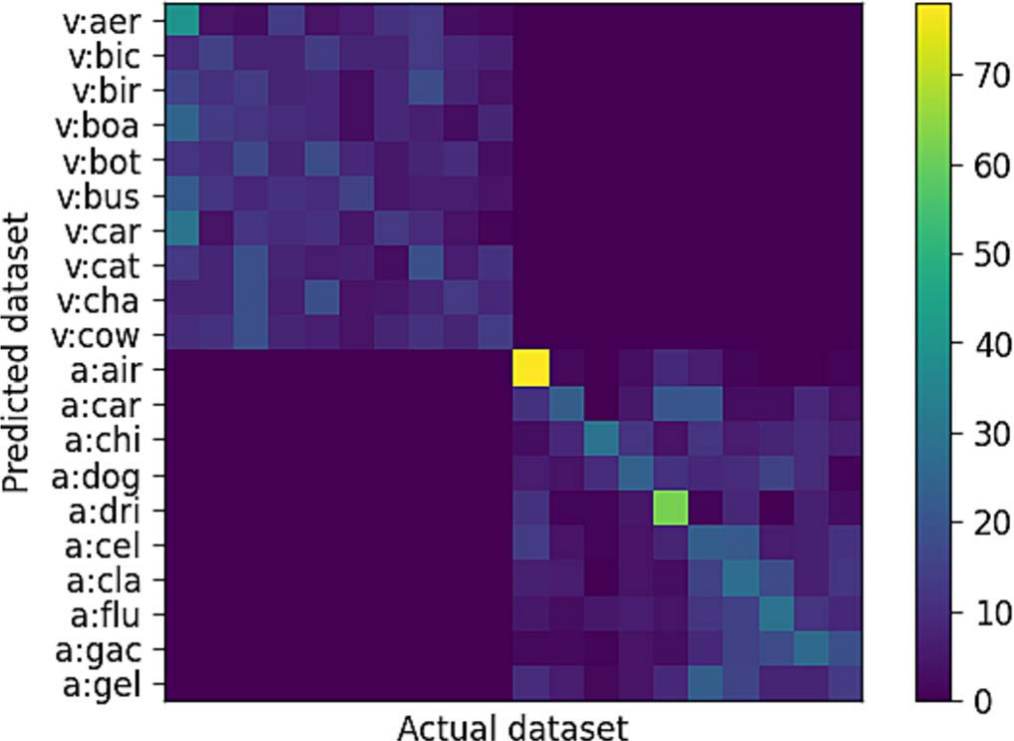
Confusion matrix of prediction of specific stimulus category using meta-representations embedding first-order networks.

The confusion matrix for the meta-classifier is shown in Fig. 4. All the diagonal elements for the meta-encoder results were above the chance level (10%). As can be seen, most of the confusion was observed within the same modality.

## Discussion

In this study, we proposed to consider meta-representations as representations of processes from the perspective of implementing the HOT of consciousness with artificial neural networks. To offer a concrete illustration, we developed "meta-networks" to embed first-order neural networks within a latent space. Through this approach, we aimed at operationalizing the concept of meta-representation. Our results show that these meta-networks are capable of differentiating networks trained on distinct sensory modalities. This highlights their ability to capture the qualitative nuances of the first-order networks. Our re-interpretation of meta-representation opens new avenues for investigating the neural correlates of these representations and their potential roles in facilitating the report of the qualitative aspects of sensory experiences in humans.

In what follows, we discuss potential implications for the neural correlates of meta-representation and the functional roles of meta-representation. Furthermore, we discuss a speculative hypothesis that qualia themselves may be considered as meta-representations, proposing a framework to capture the qualitative aspects of conscious experience from a functional perspective. We also briefly touch upon how our approach to embed processes might be used to explore the conceptual implications of the Integrated Information Theory (IIT) (Oizumi et al. 2014, Tononi et al. 2016, Albantakis et al. 2023).

### Neural correlates of meta-representation

In our discussion, we have posited that meta-representations can be understood as representations of processes, and through artificial neural networks, we showed that such meta-representations can capture the qualitative nature of stimulus modality and categories. The existence of analogous meta-representations within biological brains remains an open question, yet our approach introduces a compelling hypothesis: certain brain regions might encode the processes that transform representations from one region to another.

A potential challenge to this hypothesis arises from the fact that in biological systems, distant brain areas do not directly access the synaptic weights of each other, making the type of embedding demonstrated in our study seemingly implausible in natural brains. Nevertheless, an alternative form of embedding could be achieved through the analysis of input–output relationships across different brain regions. For instance, the meta-learning autoencoder utilizes concatenated pairs of images and labels to embed tasks (Wu et al. 2018), mirroring the embedding of a neural network trained for specific tasks. Similarly, input–output relationships in a task can be used to obtain the latent representation of the task (Lampinen and McClelland 2020). Such an approach to obtain representations of functions suggests that, without direct access to synaptic weights, the qualitative attributes of a process transforming information between two regions can still be discerned through the observation of concurrent inputs and outputs.

This insight leads to the intriguing possibility that a brain region receiving simultaneous inputs from multiple sources might learn and encode the nature of the transformations occurring between these regions. Given the interconnected networks in the brain, it is plausible that it has evolved mechanisms to map the relational patterns among these regions. This unique perspective offers new ways to inspect neurophysiological data and may deepen our understanding of the neural correlates of meta-representations.

### Functional roles of meta-representation

The incorporation of meta-representations of first-order processes creates an embedding space of neural networks where comparisons of different meta-representations are feasible in a manner similar to how semantic embeddings, like Word2Vec (Mikolov et al. 2013), map out relationships among words in natural languages. This feature enables the proposed meta-representations to support qualitative assessments of neural networks based on their functional similarities and differences.

This capability enhances cognitive flexibility and adaptability for both biological organisms and artificial systems. Meta-representations allow these systems to introspect and evaluate their own processing functions, which can improve the selection and integration of relevant processes for complex cognitive tasks such as planning, decision-making, and problem-solving. By representing the quality of sensory information processing, a system can better evaluate the meaning of the incoming sensory signals and adapt to novel situations more efficiently. Furthermore, meta-representations facilitate a deeper understanding of the relationships between different sensory modalities and experiences by allowing to discover analogies among different sensory experiences. In the context of consciousness, this ability to reflect on internal representations should be essential for reporting the contents of subjective experience in a nuanced manner.

### Connectivity patterns as the intrinsic property of networks

While our primary framework remains within HOT, our approach to the qualitative aspect of neural networks reveals an interesting link to the conceptual interpretation of the IIT. Our approach to the qualitative aspect of neural networks aligns with the conceptual interpretation of the IIT. Broadly speaking, IIT can be divided into two distinct perspectives. One is the quantitative aspect, as previously discussed as Weak IIT (Mediano et al. 2022), and another is the qualitative approach, which we introduce as **Conceptual IIT**. Both perspectives can be taken as a way to eschew theoretical commitments to the fundamental claims of Strong IIT, while facilitating empirical research motivated by IIT. (see also, Leung and Tsuchiya 2023).

Weak IIT aims to identify quantifiable empirical correlates of consciousness (Mediano et al. 2022) using more computable measures (Barrett and Seth 2011, Oizumi et al. 2016, Kitazono et al. 2018, 2020, Rosas et al. 2019, Luppi et al. 2022) or surrogate measures of integrated information, such as perturbation complexity index (e.g. Casali et al. 2013). While sharing motivations with the IIT, its emphasis is on practical measures applicable to real brain data. Unlike the strong IIT that views integrated information as identical to consciousness, the Weak IIT views integrated information as a practical measure of consciousness.

Conceptual IIT, which we advocate here, focuses on theoretical implications of IIT while avoiding the complexities of direct application of the mathematical formalisms of IIT. In the context of our current study, IIT claims that the quality of subjective experience, or “qualia”, is determined by the causal structure of the network, often expressed by the transition probability matrix (TPM) of the system (Oizumi et al. 2014, Tononi et al. 2016, Albantakis et al. 2023). Our approach to predict the sensory modality of a network only from its connectivity patterns (i.e. weight structure) is in line with this implication of IIT. However, instead of computing the structure of integrated information as designated by IIT, we used a deep learning model to directly address this prediction of IIT.

There is already empirical support for this general idea that whether stimuli are perceived as visual or auditory is determined by the intrinsic connectivity patterns from a series of experiments conducted by Sur's group. In those studies, researchers redirected retinal projections of newborn ferrets to connect with the auditory thalamus (Sharma et al. 2000, von Melchner et al. 2000). Remarkably, when inputs from the retina were directed to the auditory cortex, the neurons within this auditory region exhibited receptive fields and stimulus selectivity similar to those found in the visual cortex. They also showed that neurons in the rewired auditory cortex exhibited local connectivity patterns similar to those found in the visual cortex. Furthermore, with a carefully designed experiment, they showed that those ferrets exhibited behaviors indicating that the visual stimuli processed in the auditory cortex were perceived as visual rather than auditory (von Melchner et al. 2000). This suggests that subjective judgments of sensory modality are reflected in the specific patterns of neuronal connectivity rather than the location in the cortex. These neuroscientific findings suggest that the contribution of a specific cortical region to either visual or auditory experiences hinges on the local wiring patterns, which were shaped by the learning process of the statistical structure of the input modality.

Our current results suggest that artificial neural networks with non-modality-specific architectures learn weight parameters with distinct structures depending on the modality of training data. Since it is generally difficult to measure all connectivity in the cortex, including the weights in biological brains, artificial neural networks can be used as a surrogate model for modality-specific neural networks to test the conceptual implications of mathematical theories that are intractable to compute in biological systems.

### Why can we predict the modality neural networks were trained on?

Our results indicate that meta-representations of neural networks capture the modality-specific characteristics in the weight matrix learned by the first-order networks. One possible hypothesis is that weight matrices may have learned to gain the invariances/equivariances specific to each modality (Bronstein et al. 2017, Gerken et al. 2023). It is known that vision and audition exhibit different types of invariances. For example, in vision, the same object can be categorized as the same when the image is translated horizontally or vertically (Rao and Ruderman 1998). It also tolerates some degree of rotation and scaling. The invariances across these types of transformation are considered as specific types of symmetry. On the other hand, the same kinds of transformation may severely affect the identity of auditory events. For example, the translation across different frequencies changes the perceived contents of the auditory event, but for musical notes, transposition by an octave preserves the note’s identity, reflecting a modality-specific frequency invariance. Such differences between the two modalities can be found in the literature of studies that tried to capture independent components in natural images and sounds (Bell and Sejnowski 1997, Terashima and Okada 2012). While this remains a hypothesis at present, it will be an interesting avenue to study the invariances and equivariances embedded in the trained weight structure.

Currently, various methods to characterize the structure of weight matrices in deep neural networks are being studied in AI research (Godfrey et al. 2022, Hecht-Nielsen 1990, Navon et al. 2023, Zhou et al. 2024). Such methods may prove useful for characterizing the neural connectivity patterns underlying modality-specific architectures.

### Qualia as meta-representations

Our exploration suggests a connection between the qualitative aspects of experience and the meta-representations of first-order processes. If the brain encodes the relationships between neural network inputs and outputs, this encoding forms a meta-representation space where each point corresponds to a specific neural process. In this framework, neural networks in the brain (or filters) are situated within this space, enabling the comparison of their functionalities based on their spatial relationships in this space. This allows for the assessment of similarity between different colors, such as red and purple, or between different sensory modalities, such as vision *versus* audition. This suggests that our perception of qualities such as the redness of an object is determined by the relative positions in the meta-representation space. Notably, our framework resonates with the sensorimotor theory of consciousness (O’Regan and Noë 2001), which holds that phenomenal qualities arise from an agent’s mastery of the lawful action–sensory contingencies characteristic of each modality.

Building on this, we propose the “Qualia as Meta-representation Hypothesis”: The brain represents the input–output relationships of neural networks in a dedicated meta-representation space, which we call "qualia space." Here, the quality of our perception—qualia—is derived from the interrelations among meta-representations in this space. This qualia space representing sensory processes enables comparative analyses of experiences based on their spatial relations. Such a framework could explain how humans articulate the nuances of conscious experiences, relying on the spatial structure within this space to distinguish between different colors or sensory modalities.

Although this hypothesis implies that meta-representations are necessary to be able to report the qualitative aspects of conscious experience *via* relational comparisons to other sensory experiences, several important limitations must be acknowledged. Our current proof-of-concept focuses exclusively on process-level representations while excluding activation dynamics, and does not yet constitute a complete computational account of qualia. Future models should integrate both process parameters and activation patterns, while addressing the biological plausibility of meta-representational mechanisms in real neural systems.

Alternatively, mere presence of the intrinsic connectivity patterns within the first-order processes may be sufficient to produce conscious experience. This aligns with ongoing debates between first-order and HOT of consciousness; our approach to formulating meta-representation offers new ways to consider this debate.

## Concluding remarks

In this paper, we have discussed a possible computational implementation of the HOT of consciousness with a special focus on the concept of meta-representations as representations of processes rather than mere transformations of first-order representations. To illustrate the idea, we developed meta-networks using artificial neural networks and embedded first-order neural networks into a latent space. This approach operationalizes the notion of meta-representation within the framework of deep learning. Our results indicate that these meta-networks can distinguish between networks trained on visual and auditory datasets, thereby capturing the qualitative aspects of the networks. This conceptualization of meta-representation offers a new way to investigate the neural underpinnings of meta-representations and their potential to facilitate reporting on the qualitative aspects of sensory experiences. Finally, our findings highlight the utility of a constructive approach in refining theoretical concepts in theories of consciousness by demonstrating the feasibility of implementing theoretical concepts within artificial neural networks.

## Supplementary Material

OPEN_SCIENCE_BADGE_APPLICATION_FORM_(2)_niaf038(1)

## Data Availability

The codes used in the experiment are fully available at the following link: https://github.com/rtakatsky/metarep.
